# Injured Libyan combatant patients: both vectors and victims of multiresistance bacteria?

**DOI:** 10.3402/ljm.v8i0.20325

**Published:** 2013-02-12

**Authors:** Abdulaziz Zorgani, Hisham Ziglam

**Affiliations:** 1 Department of Medical Microbiology, Faculty of Medicine University of Tripoli, PO Box 12456, Tripoli, Libya; 2 Department of Infectious Disease, Central Hospital, Tripoli, Libya

The emergence of multidrug resistant bacteria is a global health problem (1–3), affecting the management and outcomes of a wide spectrum of infections particularly in hospitals. Resistance contributes to mortality and compromises the healthcare security of nations. Moreover, resistant pathogens are spread between countries by human travel (4–8), including the medical transfer and evacuation of combat casualties.

Over 30,000 young patients injured during the recent conflict in Libya were transferred directly or indirectly to hospitals in North Africa, the Middle East, and Europe for treatment. Many of those transferred to Europe were found to be colonized or infected with multiresistant organisms, including methicillin-resistant *Staphylococcus aureus* (MRSA), extended-spectrum β-lactamase (ESBL) – and/or *Enterobacteriaceae*, *Acinetobacter baumannii*, and *Pseudomonas aeruginosa*.

In Germany, *Klebsiella pneumoniae* harboring OXA-48, CTX-M-15, and a DHA-1 AmpC-β-lactamases was recovered from 17 Libyan injured patients along with *A. baumannii* carrying OXA-23 and NDM-1 carbapenemases. It was suggested that the bacteria with these carbapenemases were acquired while the patients were hospitalized in Libya (9). Most recently, 45 patients admitted to Danish hospitals were found to be carriers of *K. pneumonia* with OXA-48 carbapenemase; three had *A. baumannii* with OXA-23 enzyme, one had *A. baumannii* with NDM-1, and five carried MRSA. MRSA t037-III was found in two of the patients and is a rare *spa* type in Denmark, while the isolates with OXA-48-producing and NDM-1 enzymes were the first producers of these enzymes to be recorded in Denmark (10). Similarly, the first OXA-48-positive-*K. pneumoniae* to be documented in Slovenia was obtained from an injured Libyan combatant (11). The United Kingdom has also reported several isolations of OXA-48-positive *K. pneumoniae* from patients transferred for treatment following combat injuries in Libya (12). Even before these reports, the European Centre for Disease Prevention and Control (ECDC) circulated a rapid risk assessment on October 31, 2011, stating that the provision of healthcare to patients transferred from Libya to the European Union presents a high risk of introducing multiresistant bacteria (13). This, along with other ECDC risk assessments specifically relating to carbapenemase-producing *Enterobacteriaceae*, was distributed to institutions accepting Libyan patients and their microbiological laboratories (14).

Several local studies have reported widespread multiresistant organisms in Libya itself. Recently, Franka et al. found multiresistance rates exceeding 50% among Gram-negative bacilli which were recovered from screening specimens of 36 combatant patients treated at three hospitals in Tripoli, Libya (15), whilst 144 of 498 (29.8%) patients admitted to Tripoli Medical Centre with war wound–associated infections carried multiresistant *A. baumannii* (16). However, the first MDR organisms were isolated from 10 patients referred to Malta. The most perturbing being *Klebsiella pneumonia* resistant to carbapenems (KPC) of the 10 patients treated at Mater Dei Hospital in Malta (personal communication).

Despite these observation, it is difficult to estimate the true burden of antibiotic resistance in Libya: the laboratories lack a mandatory national surveillance system, have differing methods of antibiotic susceptibility testing with no mandatory quality assurance–quality control and accreditation system, and most importantly, they lack the skills, experience and the capacity to characterize multiresistant pathogens such as those with ESBLs or OXA-48 enzyme. In addition, clinicians are not aware of the challenges and risk factors associated with these organisms, with the remaining therapeutic options or with the appropriate infection control measures (17, 18). The role of health-care workers in the nosocomial transmission of MRSA has been widely discussed, following the detection of high carriage rates, varying between 11.6 and 50%, in different health-care settings, again reflecting the lack of infection control measures (19–21). Basic measures such as hospital cleanliness, unfounded hospital and community use of antibiotics, and preventative measures (e.g. hand hygiene, isolation and contact precautions) are not considered. Strategically, there is a lack of comprehensive and coherent plans and resources.

*Abdulaziz Zorgani* Department of Medical Microbiology Faculty of Medicine University of Tripoli PO Box 12456 Tripoli, Libya Email: zorgania@yahoo.com*Hisham Ziglam* Department of Infectious Disease Central Hospital Tripoli, Libya
